# Isolated congenital diaphragmatic hernia and three-year neurodevelopmental outcomes

**DOI:** 10.1038/s41390-025-03870-z

**Published:** 2025-01-23

**Authors:** Katsuaki Toyoshima, Hirosato Aoki, Kaoru Katsumata, Yoshiaki Sato, Hirosuke Inoue, Miharu Ito, Shoichiro Amari, Hidehiko Maruyama, Hitomi Arahori, Takuya Kondo, Kiyokazu Kim, Masaya Yamoto, Tomoko Saito, Hiroomi Okuyama, Noriaki Usui, Keita Terui, Kouji Nagata, on behalf of the Japanese Congenital Diaphragmatic Hernia Study Group, Katsuaki Toyoshima, Hirosato Aoki, Kaoru Katsumata, Yoshiaki Sato, Hirosuke Inoue, Miharu Ito, Shoichiro Amari, Hidehiko Maruyama, Hitomi Arahori, Takuya Kondo, Kiyokazu Kim, Masaya Yamoto, Tomoko Saito, Hiroomi Okuyama, Noriaki Usui, Keita Terui, Kouji Nagata

**Affiliations:** 1 Department of Neonatology, Kanagawa Children’s Medical Center, Yokohama, Japan; 2 Division of Neonatology, Center for Maternal-Neonatal Care, Nagoya University Hospital, Nagoya, Japan; 3 Department of Pediatrics, Graduate School of Medical Sciences, Kyushu University, Fukuoka, Japan; 4 Division of Neonatology, National Center for Child Health and Development, Tokyo, Japan; 5 Department of Pediatrics, Osaka University Graduate School of Medicine, Osaka, Japan; 6 Department of Pediatric Surgery, Graduate School of Medical Sciences, Kyushu University, Fukuoka, Japan; 7 Department of Pediatric Surgery, Kyoto Prefectural University of Medicine, Kyoto, Japan; 8 Department of Pediatric Surgery, Shizuoka Children’s Hospital, Shizuoka, Japan; 9 Department of Pediatric Surgery, Osaka University Graduate School of Medicine, Osaka, Japan; 10 Department of Pediatric Surgery, Osaka Women’s and Children’s Hospital, Osaka, Japan; 11 Division of Pediatric Surgery, Department of Surgery, Jichi Medical University, School of Medicine, Tochigi, Japan

## Abstract

**Background:**

To retrospectively investigate the developmental outcomes at 3 years of age in patients with congenital diaphragmatic hernia (CDH) using a multicenter collaborative research approach.

**Methods:**

We evaluated patients with CDH and no other malformations born between 2010 and 2016 in seven facilities in the Japanese CDH Research Group. The developmental quotient (DQ) at 3 years of age was evaluated using the Kyoto Scale of Psychological Development 2001, the most standardized scale in Japan. Factors associated with a DQ score < 85 were also analyzed.

**Results:**

Of 196 patients, developmental assessments at 3 years of age were performed in 132 patients (67%). Among these, 99 patients (75%) had a DQ score ≥ 85, 25 (19%) had a DQ score between 70 and 84, and 8 (6%) had a DQ score < 70. Multivariate analysis showed that the observed/expected lung area-to-head circumference ratio was an independent predictor of a DQ score < 85, with an adjusted odds ratio of 0.62 (95% confidence interval: 0.40–0.96; *p* = 0.03).

**Conclusion:**

Generally, isolated CDH is associated with good developmental outcomes for survivors, even after intensive care. However, there is a risk of neurodevelopmental impairment if pulmonary hypoplasia is present.

**Impact:**

- This research highlights the observed/expected lung area-to-head circumference ratio (o/e LHR) as a crucial indicator to predict neurodevelopmental outcomes in 3-year-old children diagnosed with isolated congenital diaphragmatic hernia (CDH).
- Our results provide robust evidence from a large multicenter cohort, emphasizing the importance of o/e LHR in early risk stratification and prolonged neurodevelopmental follow-up.
- These findings highlight the need for comprehensive management and tailored follow-up care in CDH patients, potentially improving clinical protocols and enhancing the quality of life and outcomes for affected children.

## Introduction

Congenital diaphragmatic hernia (CDH) is characterized by herniation of the abdominal contents into the thorax. Despite advances in neonatal intensive care and surgical management, CDH continues to cause significant mortality and morbidity.^1–3^ This serious congenital anomaly leads to herniation of abdominal organs, such as the stomach, intestines, and liver, into the thoracic cavity, resulting in pulmonary hypoplasia and pulmonary vasculature dysplasia.^4^ The combination of these conditions with persistent pulmonary hypertension of the newborn plays a pivotal role in the pathophysiology of CDH.^5–7^

Recent advances in neonatal care and surgical management have significantly improved survival rates for patients with CDH.^8,9^ However, as survival rates have increased, neurocognitive disability and decreased functional outcomes in CDH survivors have become an issue.^10,11^ The neurodevelopmental outcomes of CDH patients represent an important clinical concern.

Parents of children with CDH are often concerned about the long-term neurodevelopmental outcomes of their children. Studies have investigated developmental outcomes and predictors in CDH patients, indicating that these patients have a significant risk of cognitive and motor impairments, with prevalence rates ranging from 16% to 80%.^12–21^ The predictors of postnatal neurodevelopmental disorders (NDI) include the use of extracorporeal membrane oxygenation (ECMO) and the need for oxygen supplementation after 30 days of age, in addition to prenatal predictors, such as disease severity and gestational age at delivery. Decreased neuromuscular tone was also a predictor of NDI in several studies.^13,15,22,23^ However, these studies vary in the outcome domains that were measured, methodologies, and patients’ backgrounds (e.g., isolated CDH or non-isolated CDH, defined as CDH with additional congenital anomalies), contributing to the heterogeneity in the prevalence of NDI. Distinguishing between isolated and non-isolated CDH is crucial, as children with non-isolated CDH experience a higher incidence of NDI and have various chromosomal and structural abnormalities.^24–28^ Additionally, most studies involved univariate analysis because of the small number of patients, and even when multivariate analysis was performed, there were concerns about the validity of the analysis because of the small sample size.^29,30^ Therefore, we considered that the predictors of NDI in patients with CDH require multivariate analysis, with a sufficient number of patients and minimal heterogeneity in the patients’ backgrounds.

The aim of this study was to investigate the neurodevelopmental outcomes of patients with isolated CDH at 3 years of age using a Japanese multicenter research database, and to retrospectively examine whether disease severity is associated with NDI. We hypothesized that using a larger dataset in the analysis would clarify the impact of CDH severity on developmental outcomes, and lead to improved prognostic accuracy and family counseling.

## Methods

### Study design and patient selection

This study was part of the initiatives by the Japanese Congenital Diaphragmatic Hernia Study Group (JCDHSG) and was a retrospective cohort analysis to evaluate the neurodevelopmental outcomes at 3 years of age in children with isolated CDH. The JCDHSG database includes approximately 30% of all CDH cases in Japan and data has been collected for demographics, laboratory and imaging findings, perinatal and postnatal treatments, and outcomes since its inception in 2011.^31,32^

This study enrolled patients with CDH born between 2010 and 2016 who were registered from participating facilities, with routine developmental assessments at age 3 years. We excluded patients with congenital malformations other than CDH or known chromosomal or genetic abnormalities. Patients who died before 3 years of age or patients without developmental assessment at 3 years of age were also excluded. Strategies for perinatal management and postnatal intensive care were not standardized across centers, and instead, followed the standards at each center.

### Variables

We extracted the following detailed data from the JCDHSG database: gestational age, birth weight, 5-min Apgar score, sex, laterality of CDH, defect size of the CDH, liver-up status (defined as more than one‐third of the height of the left thoracic space occupied by the liver),^33,34^ thoracic position of the stomach,^34^ and umbilical arterial blood pH, duration of oxygen use, mechanical ventilation duration, nitric oxide therapy duration, age at surgery, length of hospital stay, use of pulmonary vasodilators, ECMO use, and home medical care requirements at discharge. Additional data regarding the prenatal diagnosis of CDH, presence of polyhydramnios, fetal hydrops, observed/expected lung-to-head ratio (o/e LHR),^35–37^ fetal treatment for CDH, place of birth, mode of delivery, multiple gestation, and prenatal steroid administration were also included. In this study, fetal ultrasound findings such as the observed/expected lung-to-head ratio (o/e LHR), liver herniation, and stomach herniation into the thoracic cavity were based on data measured at the initial assessment.

Small for gestational age was defined as less than 10% of both height and weight compared with Japanese infant standards.^38^

The position of the stomach was categorized as follows: grade 0, abdominal; grade 1, left thoracic; grade 2, less than half of the stomach herniated into the right chest; and grade 3, more than half of the stomach herniated into the right chest.^34^ Liver-up and the stomach position were only counted in patients with left CDH.

### Developmental assessment

To evaluate the neurodevelopmental outcomes, children with CDH who survived were assessed using the Kyoto Scale of Psychological Development 2001 (KSPD) at the chronological age of 36–42 months.^39,40^ The KSPD is a standardized and validated developmental test specifically designed for Japanese children. This test measures the developmental quotient (DQ) across three domains: postural-motor, cognitive-adaptive, and language-social. The overall DQ score was calculated by averaging the scores from these three categories. This follow-up protocol classifies developmental function as normal (DQ score ≥ 85), subnormal (DQ score 70–84), or delayed (DQ score < 70), with a DQ score < 70 indicating a high risk of adverse neurodevelopmental outcomes.

These variables were carefully chosen to explore their association with neurodevelopmental outcomes, specifically focusing on factors related to a DQ score < 85. We defined NDI as an overall DQ score < 85.

### Statistical analysis

We reported median (interquartile range) for continuous variables and number (percentage) for categorical variables. The analysis stratified children with CDH into two groups by the DQ score as with and without NDI. We also investigated the association between variables associated with NDI. We used the Mann–Whitney U test, and Chi-square or Fisher’s exact test for the univariate analysis. Logistic regression analysis was used to identify predictors of NDI. Among various indices that refer to disease severity, we chose o/e LHR because this is a validated indicator of disease severity^35–37^ and can be used in the prenatal period for perinatal counseling. Additionally, we selected ECMO use and tube feeding at discharge as potential confounders, in accordance with previous studies.^21,41^ *p* < 0.05 was considered statistically significant. We used IBM SPSS Statistics for Windows, version 25.0. (IBM Corp., Armonk, NY) and Excel (Microsoft 365; Microsoft Inc., Redmond, WA) for all statistical analyses.

### Ethical approval

The Ethics Committee of Kanagawa Children’s Medical Center granted approval for this study (approval number 2022-96). Owing to the study’s retrospective design and the use of de-identified data, the requirement for signed informed consent was waived. Study details were published on our institution’s website, allowing for opt-out participation. This study was performed with strict adherence to the tenets of the Declaration of Helsinki and ethical guidelines for medical and health research involving human subjects.

## Results

**Fig. 1:**
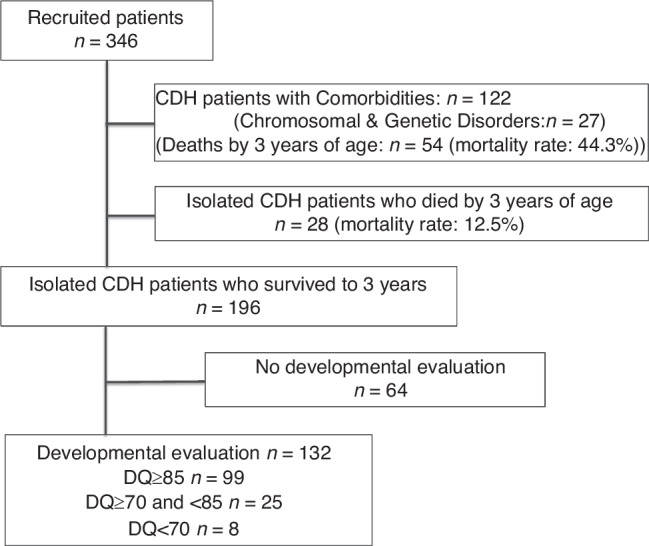
Patient flowchart. *CDH* congenital diaphragmatic hernia, *DQ* developmental quotient.

We recruited 346 CDH patients in this study. The patient inclusion flowchart is shown in Fig. 1. First, 122 patients were excluded owing to comorbid congenital malformations, including congenital heart diseases (*n* = 55), central nervous system structural abnormalities (*n* = 9), and congenital cystic adenomatoid malformation (*n* = 11). Twenty seven of them had been diagnosed with chromosomal or genetic abnormalities: Trisomy 18 (*n* = 5), Cornelia de Lange syndrome (*n* = 3), Pallister–Killian syndrome (*n* = 2), Noonan syndrome (*n* = 2), Trisomy 13 (*n* = 2), Gorlin syndrome (*n* = 1), Wolf–Hirschhorn syndrome (4p deletion syndrome) (*n* = 1), Simpson–Golabi–Behmel syndrome (*n* = 1), Opitz syndrome (*n* = 1), karyotype 47, XY, +mar (specific type if known) (*n* = 1), Fryns syndrome (*n* = 1), Apert syndrome (*n* = 1), Tetrasomy 9p (*n* = 1), chromosomal deletion 46, Y, X, del (p22.32) (*n* = 1), chromosomal translocation 46, XY, der (21)t(1;21)(q42.3;q22.2) (*n* = 1), mosaic karyotype 46, X, +mar(10)/45, X (specific type if known) (*n* = 1), Turner syndrome (*n* = 1), and partial trisomy of chromosome 14 (*n* = 1). Of the 122 CDH patients who also had congenital malformations, 54 died by the age of 3, giving a mortality rate of 44.3%.

Second, among the 224 isolated CDH patients, we excluded 28 who died before reaching 3 years of age, resulting in a mortality rate of 12.5%.

Third, we also excluded 64 patients who had not undergone developmental assessments at 3 years of age. There were no differences in background characteristics other than tube feeding at discharge between patients with and without developmental assessment (Supplemental Table). After excluding the indicated patients, 132 remained as study patients. Among them, 99 demonstrated normal developmental progress, with DQ scores ≥ 85. There were 25 children with subnormal development, with DQ scores ranging between 70 and 84, and 8 children with delayed development, with DQ scores < 70. Collectively, these results indicated that just over half of the children in the patient population had normal DQ scores, while 25% experienced some degree of developmental delay (Fig. 1).

Comparisons of patients with DQ scores ≥ 85 and < 85 are shown in Table 1.

**Table 1:** Comparison of the background characteristics of infants with congenital diaphragmatic hernia.

	DQ ≥ 85 *n* = 99	DQ < 85 *n* = 33	*p*-value
Fetal diagnosis	80 (80.8)	25 (75.8)	0.53
Fetal treatment	0 (0.0)	5 (15.6)	0.001
Fetal hydrops	0 (0.0)	0 (0.0)	NA
Oligohydramnios	14 (15.1)	8 (25.8)	0.18
Liver-up	26 (26.3)	15 (45.5)	0.04^*^
Right-sided CDH	6 (6.1)	6 (18.2)	0.07
o/e LHR	51.4 (36.5–60.4)	32.8 (28.1–38.2)	0.004^†^
Kitano criteria (Stomach position)			0.14
Grade 0: Abdominal	31 (39.2)	4 (16.7)	
Grade 1: Left thoracic	18 (22.8)	6 (25.0)	
Grade 2: Less than half of the stomach in the right thorax	19 (24.1)	7 (29.2)	
Grade 3: More than half of the stomach in the right thorax	11 (13.9)	7 (29.2)	
Antenatal steroids	4 (4.3)	1 (3.1)	>0.99
Multiple gestation	1 (1.0)	1 (3.1)	0.44
Gestational age at birth (weeks)	38.2 (37.6–38.7)	37.5 (34.7–38.4)	0.97
Birth weight (g)	2677 (2534–2954)	2569 (2310–2825)	0.37
Sex (male)	52 (52.5)	22 (66.7)	0.16
Small for gestational age	4 (4.3)	4 (12.5)	0.20
Extramural birth	20 (20.2)	9 (27.3)	0.40
Cesarian section	64 (64.6)	18 (54.5)	0.30
Umbilical cord artery blood pH	7.32 (7.28–7.34)	7.33 (7.29–7.38)	0.89
Apgar score (5-min)	6 (4–8)	2 (2–4)	0.16
Inhaled nitric oxide use	67 (68.4)	27 (81.8)	0.14
Duration of inhaled nitric oxide (days)	9 (7–11)	10 (8–12)	0.05
Duration of oxygen administration (days)	24 (17–38)	23 (20–39)	0.14
Duration of mechanical ventilation (days)	17 (9–22)	20 (18–22)	0.02
Age at surgery (days)	2 (2–3)	2 (1–2)	0.47
Defect size in the diaphragm			0.29
<10%	20 (21.5)	7 (25.9)	
10–49%	51 (54.8)	10 (37.0)	
50–89%	12 (12.9)	7 (25.9)	
>90%	10 (10.8)	3 (11.1)	
Patch closure (vs direct closure)	23 (23.2)	12 (41.3)	0.05
Duration of hospitalization (days)	40 (33–54)	77 (60–90)	<0.001
Pulmonary vasodilator use	4 (4.1)	7 (21.2)	0.006
ECMO use	2 (2.0)	4 (12.1)	0.03
Tube feeding at discharge	1 (1.0)	4 (12.1)	0.01
Respiratory support at discharge	0 (0.0)	5 (15.2)	0.001
Tracheostomy at discharge	0 (0.0)	1 (3.0)	0.25

Values are shown as number (percentage) or median (interquartile range).

^*^. ^†^*CDH* congenital diaphragmatic hernia, *DQ* developmental quotient, *ECMO* extracorporeal membrane oxygenation, *o/e LHR* observed/expected lung area-to-head circumference ratio.

The timing of the fetal ultrasound measurements for the o/e LHR, liver herniation, and stomach herniation into the thoracic cavity was at a median (interquartile range) gestational age of 30.6 weeks (27.2–34.0 weeks). Fetal treatment (*p* = 0.001), o/e LHR (*p* = 0.04), duration of mechanical ventilation (*p* = 0.02), duration of hospitalization (*p* < 0.001), pulmonary vasodilator use (*p* = 0.006), ECMO use (*p* = 0.03), tube feeding at discharge (*p* = 0.01), and respiratory support at discharge (*p* = 0.001) differed significantly between the two groups. In contrast, no significant differences were seen between the groups for best preductal arterial partial pressure of oxygen within 24 h, duration of oxygen use, duration of nitric oxide use, ECMO use, small for gestational age status, sex, prenatal diagnosis, polyhydramnios, prenatal steroid administration, extramural birth, cesarean delivery, and multiple gestation (Table 1).

For the multivariate analysis, variables were selected in accordance with findings from previous research and the results of our univariate analysis, specifically focusing on o/e LHR, ECMO use, and tube feeding. In the multivariate logistic regression analysis, o/e LHR remained a predictor of NDI (adjusted odds ratio: 0.62; 95% confidence interval (CI): 0.40–0.96; *p* = 0.03). ECMO use and tube feeding at discharge, although not statistically significant, indicated a trend toward an association with neurodevelopmental outcomes (adjusted odds ratio (OR) for ECMO use: 4.17, 95% CI: 0.33–52.43; *p* = 0.27 and adjusted OR for tube feeding at discharge: 3.74, 95% CI: 0.28–49.26; *p* = 0.32) (Table 2).

**Table 2:** Predictors of neurodevelopmental impairment in infants with congenital diaphragmatic hernia.

	Unadjusted OR (95% CI)	*p*-value	Adjusted OR (95% CI)	*p*-value
o/e LHR (per 10%)	0.56 (0.37–0.85)	0.007	0.62 (0.40–0.96)	0.03
ECMO	6.69 (1.17–38.39)	0.033	4.17 (0.33–52.43)	0.27
Tube feeding at discharge	13.52 (1.4–125.7)	0.02	3.74 (0.28–49.26)	0.32

*ECMO* extracorporeal membrane oxygenation, *o/e LHR* observed/expected lung area-to-head circumference ratio, *OR* odds ratio, *95% CI* 95% confidence interval.

**Fig. 2:**
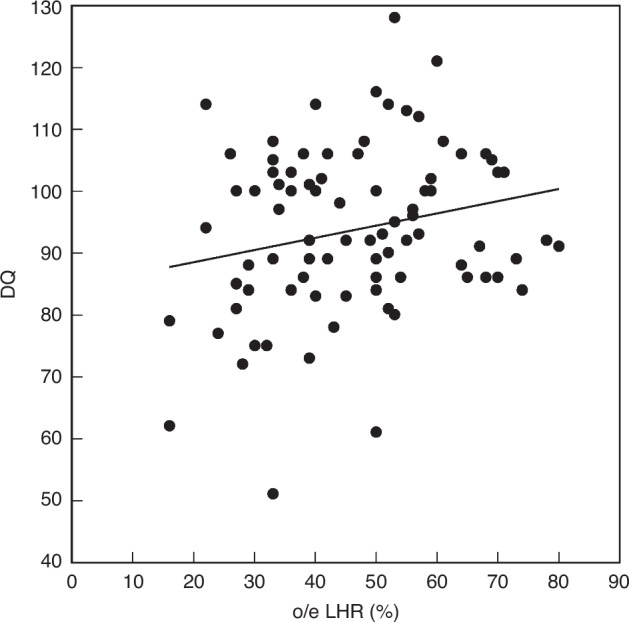
Correlation between o/e LHR and DQ score. The o/e LHR was weakly correlated with the DQ score (DQ = 0.20 × o/e LHR + 84.6 ; R^2^ = 0.05; *p* = 0.04). *o/e LHR* observed/expected lung area-to-head circumference ratio, *DQ* developmental quotient

The o/e LHR was weakly correlated with the DQ score (DQ = 0.20 × o/e LHR + 84.6 ; R^2^ = 0.05; *p* = 0.04) (Fig. 2).

## Discussion

In this multicenter study, we used the extensive JCDHSG database to examine neurodevelopmental outcomes in children with isolated CDH at 3 years of age. The strengths of this study are the unprecedented number of included patients, and the use of a rigorous multivariate analysis methodology. Because CDH is associated with a variety of genetic disorders and comorbidities, limiting the database to cases without comorbidities or minor malformations allowed us to determine neurodevelopmental outcomes in isolated CDH. Our analysis of 132 cases confirmed that 75% of the infants achieved a DQ score ≥ 85. This confirms the efficacy of our treatment for CDH, a serious neonatal condition that often requires intensive and surgical intervention in the neonatal period. Notably, despite the complexities associated with the management of CDH, a significant proportion of children with isolated CDH were able to achieve developmental milestones with minimal neurological sequelae, underscoring the effectiveness of current treatments in saving lives while preserving quality of life. However, approximately 17% of the children had borderline development and developmental delays, with DQ scores < 85, similar to results in previous studies that examined several factors that predict developmental delays.^12–28^ In this study, we identified factors associated with the need for developmental support and demonstrated that 17% of cases with isolated CDH required such support, underscoring its prevalence in a substantial cohort.

In the multivariate analysis, we highlighted the importance of o/e LHR as an independent predictor of neurodevelopmental outcomes in children with CDH at 3 years of age. We also identified ECMO use and tube feeding at discharge as future research questions because of their potential impact on developmental outcomes.

In the present study, o/e LHR emerged as a significant predictor of developmental outcomes, with an adjusted OR of 0.95 (95% CI: 0.91–0.99; *p* = 0.03), underscoring its utility in early risk stratification.

In the past 10 years, numerous studies have highlighted the long-term neurodevelopmental outcomes in survivors of CDH,^10,11,42^ indicating a significant risk for neurodevelopmental impairment in these individuals.^11,42,43^ Various risk factors contribute to adverse neurodevelopmental outcomes in CDH survivors. These include pulmonary hypoplasia, intrathoracic liver position, requirement for ECMO, right-sided CDH, patch repair, extended oxygen supplementation, and the need for invasive ventilation, among others.^41,42,44,45^ In children with isolated CDH, herniation of abdominal organs into the thoracic cavity during crucial stages of pulmonary development leads to pulmonary hypoplasia. The herniated organs not only compress the ipsilateral lung but also cause mediastinal shift, which can exert additional pressure on the contralateral lung. In severe cases, this bilateral pulmonary hypoplasia becomes life-threatening. To predict the severity of pulmonary hypoplasia prenatally and, consequently, postnatal survival, clinicians use imaging techniques that provide indirect measurements of fetal lung volume. Two widely recognized predictors of postnatal survival are the LHR and the o/e LHR, both of which are used globally.^36,46^ While several studies have linked a low LHR^43,44,47^ or intrathoracic liver position^16,41^ with adverse neurodevelopmental outcomes, other research has not found a significant association between these factors and neurodevelopmental outcomes.^11,23,43^ In our study, we found that o/e LHR, which is an indicator of lung hypoplasia, was a predicter of neurodevelopmental outcomes at 3 years of age in isolated CDH. This aligns with the physiological implications of reduced pulmonary function impacting overall developmental milestones. Furthermore, the o/e LHR correlated with the DQ score.

In this study, we showed that a low o/e LHR not only predicts mortality, but also has a significant impact on neurological outcomes. A low o/e LHR indicates fetal lung hypoplasia requiring prolonged postnatal interventions, such as mechanical ventilation and sedation. These intensive treatments, especially when prolonged, are associated with complex effects on neurodevelopment. Consequently, a low o/e LHR should be considered a predictor of adverse neurological and developmental outcomes, suggesting the need for a comprehensive approach to the management and follow-up of affected neonates.

A comparison of our findings with the existing literature highlights unique insights into the predictors of developmental outcomes in CDH survivors. Our rigorous multivariate analysis, which incorporated a broader set of variables compared with previous studies, provides a comprehensive understanding of the factors influencing neurodevelopmental outcomes in this population.

Although ECMO use and tube feeding at discharge did not achieve statistical significance when comparing the two patient groups, there was a trend towards an association with poorer neurodevelopmental outcomes. From infancy through school age, normal cognition scores have been reported in CDH survivors, with normal to mildly delayed overall cognitive and language-development scores at preschool age.^16,19–21,41,48,49^ ECMO use is an independent predictor of impaired mental development.^21,41,43^ Our findings were similar and indicate the critical impact of pulmonary hypoplasia and other prenatal factors on long-term outcomes. Notably, our study reinforces the role of ECMO and tube feeding at discharge, which, while not reaching statistical significance, showed trends toward an association with poorer outcomes. These results suggest potential areas for improving clinical protocols and intervention strategies.

Tube feeding was significantly more common in patients who did not vs did undergo developmental evaluation, as shown in Table 1. The reasons for the need for tube feeding are unknown and may include developmental delay, which makes developmental testing difficult, and a genetic disorder (non-isolated CDH) that has not been definitively diagnosed. Further research is needed to clarify the factors that lead to the need for tube feeding in cases of isolated CDH.

### Study limitations

While this study provides important insights into the neurodevelopmental outcomes of children with isolated CDH, several limitations must be acknowledged. First, 132 patients underwent developmental assessment among infants with isolated CDH, and 64 did not. However, the excluded group was not simply a skewed group. Prior to the analysis, we compared variables between the study patients (*n* = 132) and the excluded 64 patients who had not undergone developmental assessment. Only tube feeding at discharge was significantly higher in the excluded vs included patients (*p* = 0.007); other variables did not differ between the two groups (Supplemental Table). Second, we assessed the patients in this study using the KSPD, a developmental assessment widely used in Japan, which may complicate international comparisons. However, the KSPD has been validated and correlates highly with Bayley-III developmental test scores. The KSPD is widely used in the developmental assessment of preterm infants in Japan.^39,50^; therefore, the results of this study can be generalized internationally.

## Conclusions

Our study, with its robust methodology and high number of included patients, identified o/e LHR as a significant predictor of neurodevelopmental outcomes in children with isolated CDH. The implications of ECMO use and tube feeding at discharge regarding long-term development warrant further exploration. Our findings provide valuable insights into ongoing efforts aimed at enhancing the quality of life and outcomes for CDH patients.

## Supplementary information

Supplemental Table

## Data Availability

The data underpinning the findings of this research are available from the corresponding author, K Toyoshima on reasonable request.
